# Improving access to primary health care through financial innovation in rural China: a quasi-experimental synthetic difference-in-differences approach

**DOI:** 10.1186/s12875-024-02450-0

**Published:** 2024-06-01

**Authors:** Zhi Zeng, Yunmei Luo, Wenjuan Tao, Ruiling Zhang, Bo Zeng, Jianhong Yao, Wei Zhang

**Affiliations:** 1 Institute of Hospital Management, West China Hospital, Sichuan University, Chengdu, Sichuan China; 2 Office of Policy Research, Chinese Center for Disease Control and Prevention & Chinese Academy of Preventive Medicine, Beijing, China; 3 Chinese Academy of Medical Sciences & Peking Union Medical College, No.9, Dongdan Santiao, Beijing, 100730 China; 4 West China Biomedical Big Data Center, West China Hospital, Sichuan University, No.37, Guoxue Xiang, Chengdu, 610041 Sichuan China; 5 Med-X Center for Informatics, Sichuan University, Chengdu, Sichuan China

**Keywords:** Health financing, Pooled funds, Primary health care, Rural Health, Universal health coverage, Synthetic difference-in-differences

## Abstract

**Background:**

Inadequate financing constrains primary healthcare (PHC) capacity in many low- and middle-income countries, particularly in rural areas. This study evaluates an innovative PHC financing reform in rural China that aimed to improve access to healthcare services through supply-side integration and the establishment of a designated PHC fund.

**Methods:**

We employed a quasi-experimental synthetic difference-in-differences (SDID) approach to analyze county-level panel data from Chongqing Province, China, spanning from 2009 to 2018. The study compared the impact of the reform on PHC access and per capita health expenditures in Pengshui County with 37 other control counties (districts). We assessed the reform’s impact on two key outcomes: the share of outpatient visits at PHC facilities and per capita total PHC expenditure.

**Results:**

The reform led to a significant increase in the share of outpatient visits at PHC facilities (14.92% points; 95% CI: 6.59–23.24) and an increase in per capita total PHC expenditure (87.30 CNY; 95% CI: 3.71-170.88) in Pengshui County compared to the synthetic control. These effects were robust across alternative model specifications and increased in magnitude over time, highlighting the effectiveness of the integrated financing model in enhancing PHC capacity and access in rural China.

**Conclusions:**

This research presents compelling evidence demonstrating that horizontal integration in PHC financing significantly improved utilization and resource allocation in rural primary care settings in China. This reform serves as a pivotal model for resource-limited environments, demonstrating how supply-side financing integration can bolster PHC and facilitate progress toward universal health coverage. The findings underscore the importance of sustainable financing mechanisms and the need for policy commitment to achieve equitable healthcare access.

**Supplementary Information:**

The online version contains supplementary material available at 10.1186/s12875-024-02450-0.

## Background

Adequate and sustainable health financing is indispensable for the global attainment of universal health coverage (UHC) and the fortification of primary health care (PHC) systems [1]. PHC stands as the linchpin for establishing more equitable, efficient, and resilient health systems, with countries prioritizing PHC to realize healthier populations at reduced costs [2]. However, health financing in numerous low- and middle-income countries (LMICs) grapples with constraints like limited funding, fragmentation across schemes, and an inclination toward urban-focused curative care [3]. Particularly, rural PHC contends with underinvestment, leading to significant access, quality, and financial protection gaps. The post-COVID budgetary constraints have further exacerbated the decrease in health allocations in LMICs, emphasizing the urgent need for well-funded PHC [4].

The World Health Organization (WHO) delineates three core functions of health financing: (i) revenue raising; (ii) pooling; and (iii) purchasing [5]. While revenue raising and purchasing have received considerable attention, the role of pooling arrangements in facilitating universal coverage has received less scrutiny, especially from the supply-side perspective. Previous studies across various LMICs have explored trends in catastrophic and impoverishing health expenditures associated with different demand-side health financing schemes. Specifically, these studies have focused on evaluating the financial risk protection effects of pooling on the demand side [6–11]. However, supply-side fragmentation also contributes to inefficiency and inequity [11]. Empirical evidence on pooling and PHC access remains scarce, particularly for rural, resource-limited settings. Notably, supply-side efforts to diminish fragmentation via integrated financing mechanisms warrant increased focus.

In China, the government predominantly operates the PHC system, which forms the foundational tier of the three-tier healthcare delivery system, offering general clinical care and essential public health services [12]. China’s rural health system has long been challenged by fragmentation and underfunding [13, 14]. Despite substantial reforms in recent years, the country’s rural primary healthcare still confronts systemic challenges that impede both access and quality [15, 16]. Notwithstanding the policy focus on reinforcing PHC post-2009 national healthcare reforms in China, issues like limited funding and a lack of qualified personnel continue, exacerbating geographic and economic disparities in healthcare access [12]. Furthermore, China’s healthcare services currently face major hurdles, particularly with fragmented delivery systems that are predominantly hospital-centric and treatment-focused [16]. Additionally, the health system continues to exhibit fragmentation across various insurance schemes and levels of care, compounded by ambiguous referral processes. There is an increasing trend of patients in China bypassing primary care and directly accessing treatment at more advanced hospital facilities [17, 18].

To address the imbalanced distribution of medical resources and redirect patient flow to primary care facilities, some local governments have piloted the integration of PHC providers and hospitals [19]. Recent policy initiatives attempt to promote PHC-based integrated care by linking primary care centres with higher-level hospitals through what is termed “vertical integration” [20]. However, most pilots remain hospital-centric. Few reforms directly channel financing to frontline rural providers [21]. Pengshui County, a financially disadvantaged rural region in China, implemented an innovative horizontal integration and financing mechanisms reform to improve local PHC service delivery following China’s 2009 national healthcare reforms. Early evidence suggests that the Pengshui model strengthened rural PHC capacity, utilization, and outcomes [22, 23].

Evaluating the effects of these health financing policy innovations is vital for informed policymaking, although it presents methodological challenges in the context of localized interventions. Synthetic controls are useful for estimating single-unit effects; however, biases may occur if control units do not share similar trends prior to the intervention [24]. The novel synthetic difference-in-differences (SDID) estimator merges the synthetic control method (SCM) with difference-in-differences (DID) approaches, offering a more robust causal analysis even in scenarios with a single treated unit [25].

In this study, we aim to rigorously evaluate the impact of an innovative PHC financing reform in Pengshui County, focusing on its effectiveness in improving access to healthcare services in a rural setting. This research yields three substantial contributions. First, it provides empirical evidence and lessons for resource-constrained settings to enhance the sustainability and effectiveness of their PHC financing mechanisms. Second, it illustrates the utility of the synthetic SDID approach in empirically assessing health financing pilot reforms in scenarios where experimental designs are impractical. Third, from a supply-side perspective, it offers experiential insights into the practice of horizontal health financing reform in underdeveloped regions. Consequently, the pragmatic evidence is applicable to rural areas in China and may significantly contribute to the design, implementation, and evaluation of PHC-related pooling policies in most LMICs.

## Methods

### Study setting

Pengshui County, located in the mountainous southeast region of Chongqing, China, had approximately 0.5 million residents, mostly rural, in 2018. Historically, it was categorized as economically underprivileged at the national level (refer to Supplementary Fig. 1) [26]. The local PHC system comprises 36 township health centres in rural areas and 4 community health service centres in urban settings, serving as the foundation of the three-tiered healthcare delivery system. The PHC institutions are typically staffed by a mix of general practitioners, nurses, and public health workers, and offer a range of basic medical services, including outpatient care, inpatient care for common diseases, minor surgeries, and public health services. These providers play a vital role in ensuring rural populations, especially those in remote and underserved regions, have access to essential healthcare services.

Being one of the poorest counties in western China, Pengshui’s medical supply system frequently suffered from inadequate local government funding and insufficient medical resources. Consequently, shortages of professional medical staff and facilities, inadequate treatment quality, frequent stockouts, suboptimal prescription practices, and poor medication usage prevailed, and most residents opted to bypass PHC centers and sought healthcare directly from higher-level hospitals instead of utilizing distrusted PHC centres nearest to their homes. In 2009, the proportion of outpatients seeking first-contact care in rural community or township health centers decreased to 29% (522,700 out of 1,817,600) [22, 23]. To solve these problems, Pengshui’s government initiated significant PHC reform, concentrating on horizontal integration and financing since 2019.

### Implementation

The reform’s key interventions included: Firstly, 40 government-run township and community health centres were consolidated into a “Primary Health Care Institution Group” (PHCIG), governed centrally. The Commission of Primary Health Care (CPHC) was established to manage the group. Secondly, in 2012, a collective “PHC Fund” was established, financed through government subsidies and mandatory contributions from PHC institutions’ revenues. This substantial fund supported investments in infrastructure, equipment, training, and performance-based remuneration. Thirdly, a Rural Health Management Centre (RHMC), encompassing Health Accounting and a Centralized Drug Purchasing Centre, was set up to oversee investment planning, manage the pooled fund, procure medications, and monitor performance.

**Fig. 1:**
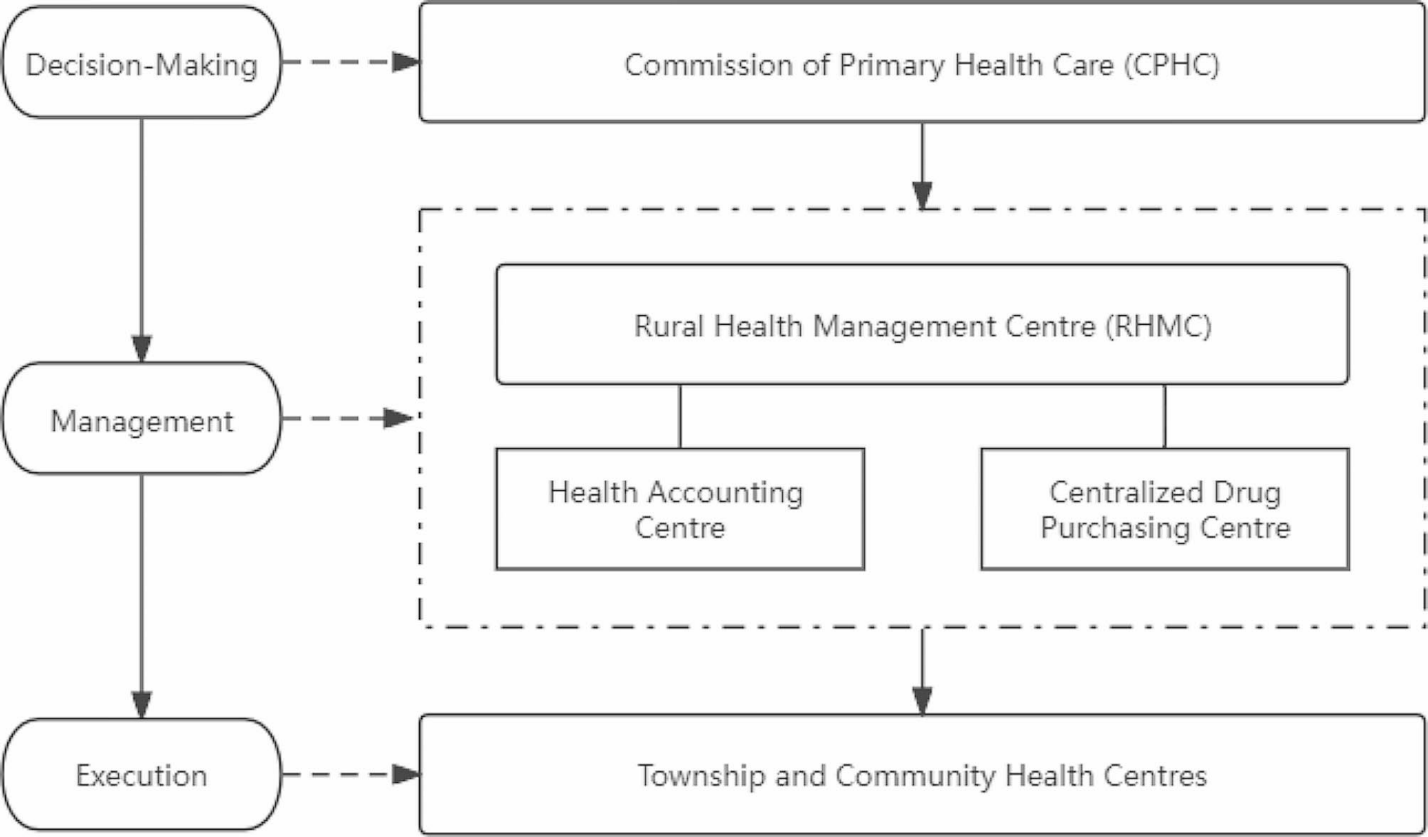
The governance structure of primary health care institution group

The governance structure of PHCIG (Fig. 1) was designed to ensure transparency, efficiency, and accountability in the allocation and utilization of resources. The CPHC, as the decision-making body, is responsible for setting the overall strategy and priorities for the allocation of the PHC funds. The RHMC, under the guidance of the CPHC, is in charge of the day-to-day management of the funds, including planning, budgeting, and monitoring the use of the funds. The RHMC conducts regular assessments of the infrastructure, equipment, and workforce needs of each PHC institution within the PHCIG. Based on these assessments, the RHMC develops an annual investment plan, which is then reviewed and approved by the CPHC. In addition, the centralized procurement approach at the PHC group level is designed to improve the efficiency and cost-effectiveness of drug purchasing for PHC facilities within Pengshui County, potentially offering advantages in terms of economies of scale and bargaining power. Moreover, to ensure transparency and accountability in the use of the PHC funds, the RHMC implements a robust monitoring and evaluation system. This includes regular financial audits, performance assessments, and reporting to the CPHC and local government authorities.

**Fig. 2:**
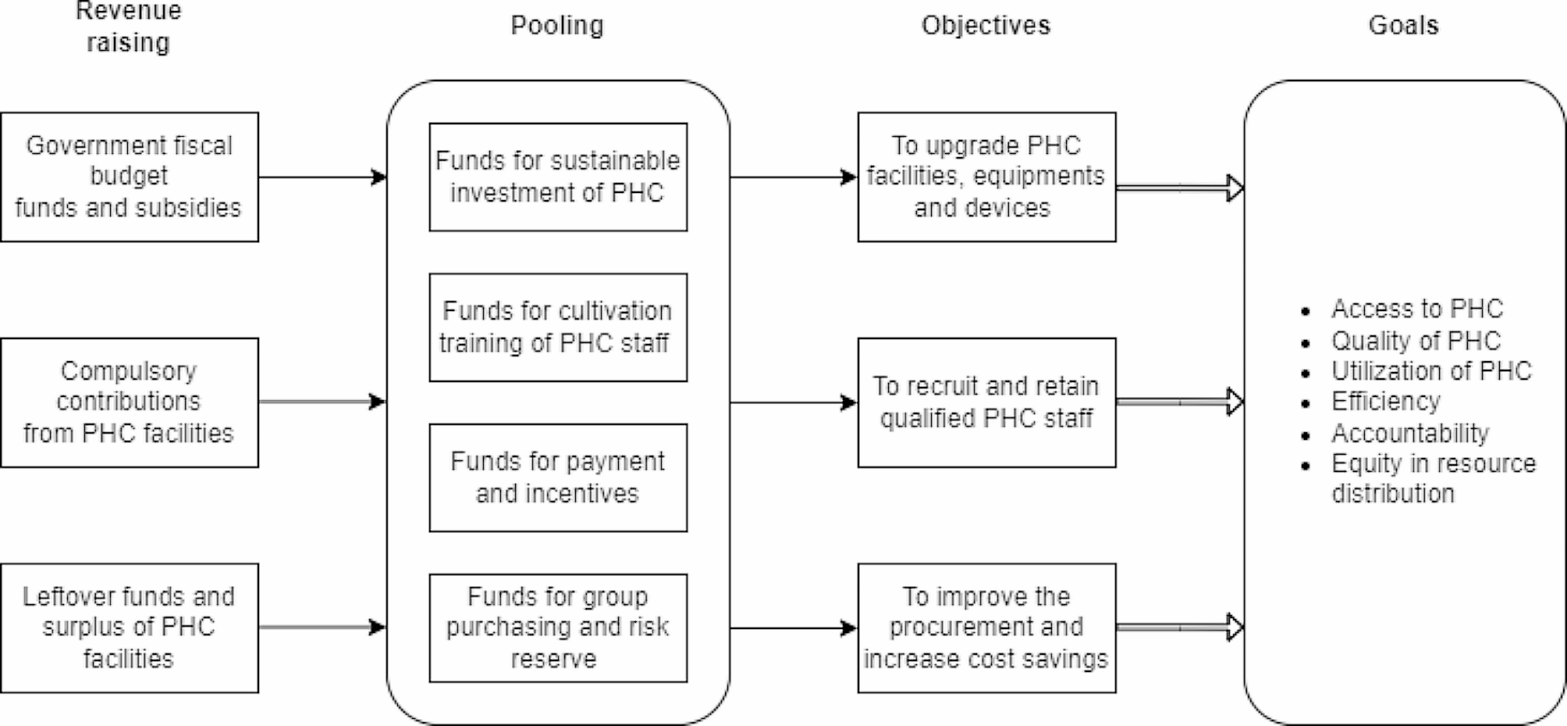
Flow of pengshui’s PHC funds. Notes: PHC = Primary Health Care; “→” refers to the flow of funds

The PHC fund, derived through horizontal integration (see Fig. 2), consists of multiple sources: (i) Government fiscal allocations and subsidies, ensuring stable financial backing for PHC investments. (ii) Mandatory contributions from PHC institutions, constituting 10% of their total and net medical revenue (the latter being total revenue minus drug costs). This mechanism reallocates funds from wealthier to less developed PHC facilities. (iii) Residual funds and surpluses from preceding years, facilitating the accumulation and rollover of resources across fiscal periods. Providers can request additional resources from the fund for locally tailored investments with interest-free repayment. Performance monitoring has been established to oversee these allocations, thereby enhancing transparency. Utilizing the WHO’s health funding strategy guidelines [3], Tables 1 and 2 outline the characteristics and impacts, while Fig. 2 illustrates the allocation of pooled PHC funds.

**Table 1:** Key strategies of financial reform in Pengshui County, Chongqing, China

Dimensions	Key implementation strategies	Contextual factors
Revenue raising	Compulsory contributions from each PHC provider’s revenue.^1^ Carry over previous years’ surplus funds. Increasing fiscal budget allocation.	Limited health investments. Fragmented financing channels constrained PHC funding.
Pooling	Merging fragmented pools into an integrated fund. Cross-subsidization to sustain less developed area PHC facilities. Supporting medical professionals’ training and income guarantees.	Redistribution based on needs enables equity. Health workforce deficits due to low compensation hindered recruitment and retention.
Purchasing	Centralized procurement of drugs and equipment. Performance incentives without income ceilings. Strategic purchasing guided by priorities.	Lack of strategic purchasing.
Financial management	Unified management account. Streamlining processes and financial oversight. Strengthening auditing of fund utilization.	Clear rules on pooling governance and auditing encourage participation.

Notes: PHC = Primary Health Care

1. Proportional financing mechanism, whereby each PHC facility contributes the same percentage of income

**Table 2:** Illustration of pooling arrangements in Pengshui County, Chongqing, China

Type of pooling arrangements	Effects on pooling objectives	Type of funds transfer
Compulsory contributions	Increase the size of the pool, and improve redistributive capacity	From organizational assets to collective funding
Merging pools	Increase the size and diversity of the pool, and reduce fragmentation	From a single pool to a unified group pool
Cross-subsidization	Improve redistributive capacity and equity	From better-resource PHC providers to underdeveloped facilities
Harmonization across pools	Improve redistributive capacity and efficiency	From inactive funds to strategic investments with system-wide priorities

Notes: PHC = Primary Health Care

The allocation of the PHC funds follows a needs-based approach, with priority given to underserved areas and facilities with the greatest resource gaps. The governance structure and allocation process of the PHC funds are designed to ensure that resources are directed to where they are needed most and that the funds are used efficiently and effectively to improve the quality and accessibility of PHC services in Pengshui County. Additionally, it allowed for strategic procurement and investments aligned with grassroots healthcare priorities. This novel funding model transformed fragmented investments into a systematic resource allocation process [27], pivotal for sustainable investment in underserved rural regions [4]. Since its establishment in 2012, the PHC fund has amassed over 400 million yuan [22]. The consistent funding from the PHC fund played a critical role in the successful implementation of the reform, enabling the targeted improvement of PHC infrastructure, equipment, and workforce.

### Study design and hypothesis

Building upon the aforementioned background, our study employs a quasi-experimental research design to elucidate the causal relationship between the implementation of the financial innovation policy and the accessibility of PHC services in Pengshui County. In this design, Pengshui serves as the singular treated unit, while the other 37 counties (districts) in Chongqing act as control entities. Given that the horizontal integration and financing reform officially commenced in 2012, we designate the pre-reform period as 2009–2011 and the post-reform period as 2012–2018.

Therefore, we posit the following specific hypotheses:

#### Hypothesis 1

The horizontal integration and financing reform prove effective in guiding residents to seek healthcare services within PHC facilities.

#### Hypothesis 2

The financial reform enhances health expenditures towards township and community health service centres.

### Data and variables

Data from Chongqing provincial statistics yearbooks covering 2009 to 2018 were used to construct a balanced county-level panel dataset, encompassing Pengshui County and 37 other counties (districts).

Following the PHC measurement framework and indicators provided by the WHO [28], we analyzed two key outcome variables related to PHC access and utilization:

(i) **Share of outpatient visits at PHC facilities**: This metric indicates the availability, readiness, and patient utilization of PHC services. It can also serve as an indirect proxy for the perceived quality and acceptability of PHC services. A higher share of outpatient visits at PHC facilities may suggest that these facilities are sufficiently equipped and staffed to meet patients’ needs and that patients have a certain level of trust in the care provided.
(ii) **Per capita total PHC expenditure**: This metric assesses the total healthcare expenditure within the PHC sector. Importantly, the per capita total PHC expenditure measure, encompassing both individual out-of-pocket payments and the corresponding health insurance reimbursements, offers a comprehensive overview of the financial resources allocated to the PHC system. Therefore, this metric serves as a vital indicator of economic accessibility to primary care services.

### Statistical analysis

The design leverages a synthetic difference-in-differences (SDID) approach, which combines elements of the synthetic control method (SCM) and difference-in-differences (DID) approaches [25]. The SDID approach offers several advantages over traditional DID and SCM, particularly in the context of evaluating localized interventions with a single treated unit. Unlike DID, which relies on the parallel trends assumption, SDID can accommodate scenarios where the treated and control units have different pre-intervention trends by re-weighting the control units to create a more suitable counterfactual. Moreover, by incorporating unit-specific time weights, SDID allows for greater flexibility in modeling the temporal dynamics of the intervention’s impact.

Compared to SCM, SDID is more robust to violations of the convex hull assumption, which requires that the treated unit’s outcomes can be approximated by a weighted combination of the control unit’s outcomes. By combining SCM with a DID framework, SDID can mitigate bias arising from imperfect pre-treatment fit between the treated unit and its synthetic control. Furthermore, SDID allows for valid statistical inference even when there is only a single treated unit, making it particularly valuable for evaluating reforms implemented at a local or regional level, such as the Pengshui model.

The SDID approach involves several key steps: First, we estimate weights for the control units to ensure that pre-treatment trends between the treated and control groups are parallel. This alignment is crucial for the validity of the counterfactual comparison. Next, we construct a synthetic control group for Pengshui County by re-weighting the control units. This synthetic control group is designed to mimic the pre-treatment characteristics of Pengshui as closely as possible. We then introduce time weights to account for differences in the timing and duration of the intervention across units. This step helps to control for time-varying factors that could affect the outcomes. Finally, we estimate a two-way fixed effects regression model using the synthetic control and the actual outcomes of Pengshui County. The model includes fixed effects for both units and time, which helps to control for unobserved, constant differences between units and time-invariant factors [24, 25, 29, 30].

In our analysis, we incorporate several covariates, including per capita government health expenditure, population size, urbanization rate, and GDP per capita. These factors are considered to enhance the precision and reliability of the model’s estimates. The primary outcome measures are the share of outpatient visits at PHC facilities and the per capita total PHC expenditure. These indicators reflect the utilization of PHC services and the allocation of resources to these services, respectively.

We employed the placebo method to estimate the standard errors of the treatment effect estimates, accounting for potential heteroskedasticity in the regression model. The placebo method was chosen over alternatives such as bootstrapping or jackknifing due to its ability to provide robust inference in the presence of serial correlation within units, which is a common challenge in panel data settings [31]. This approach helps ensure that our inference is valid despite the potential for within-unit correlations over time [24, 25]. The statistical analysis is conducted using Stata16 software (Stata Corporation, 2016).

1 $$argmi{n_{\mu ,\alpha ,\beta }}\tau _{i = 1}^{38}\tau _{t = 1}^{10}{\left( {{X_{it}} - \mu - {\alpha _i} - {\beta _t} - {D_{it}}\tau } \right)^2}{w_i}{\lambda _t}$$

where *µ* is a constant, *α*_*i*_ is a unit-fixed effect and β_*t*_ is a time-fixed effect. The dependent variable *X*_*it*_ denotes variables in county *i* = 1, …, 38, and year *t* = 1, … ,10, referring to the year 2009, …,2018. Pengshui is the last county (*i* = 38). Since the pooling arrangement was introduced in the year 2012(*t* = 4), the treatment indicator is defined as:

2 $${D_{it}} = \left\{ {\begin{array}{*{20}{c}}1&{if\;\;i = 38\;and\;t \geqslant 4} \\ 0&{else.} \end{array}} \right.$$

The main parameter of interest is *τ*, which captures the impact of the pooling reform on PHC access and utilization. *w*_*i*_ and *λ*_*t*_ are county and time weights respectively.

By employing the SDID approach, our study aims to provide a more accurate and reliable estimate of the impact of the financial innovation policy on PHC services in Pengshui County. This methodological rigor is essential for drawing valid conclusions about the effectiveness of the intervention and its potential as a model for other settings.

## Results

### Descriptive analysis

The summary statistics and variables used are listed in Table 3. The optimized SDID weights for the 37 control counties are evenly distributed for both outcome variables. Thereafter, we discuss the estimates of the impact of the financial reform on PHC access and utilization.

**Table 3:** Descriptive statistics

Variable	(1)	(2)	(3)	(4)	(5)
	*N*	Mean	SD	Min	Max
Share of outpatient visits at PHC facilities (%)	380	25.48	10.00	5.09	72.12
Per capita total PHC expenditure (CNY)	380	159.83	65.30	15.32	429.44
Per capita government health spending on PHC (CNY)	380	108.92	61.81	0.29	287.16
Population (10,000)	380	78.30	32.85	18.43	166.17
Urbanization rate (%)	380	53.97	21.01	21.60	100.00
GDP per capita (CNY)	380	41,682	25,658	7,056	18,2540

Notes: PHC = Primary Health Care, GDP = Gross Domestic Product. “Per capita total PHC expenditure” includes individual out-of-pocket payments and health insurance reimbursements, representing the total financial burden on individuals. “Per capita government health spending on PHC” denotes the direct fiscal investment by the government in PHC institutions, excluding health insurance reimbursements. In our analysis, we control for the latter to isolate the effects of the total expenditure on PHC utilization and financial accessibility

### Synthetic difference-in-differences estimation

#### Trends in PHC outpatients’ market share

**Fig. 3:**
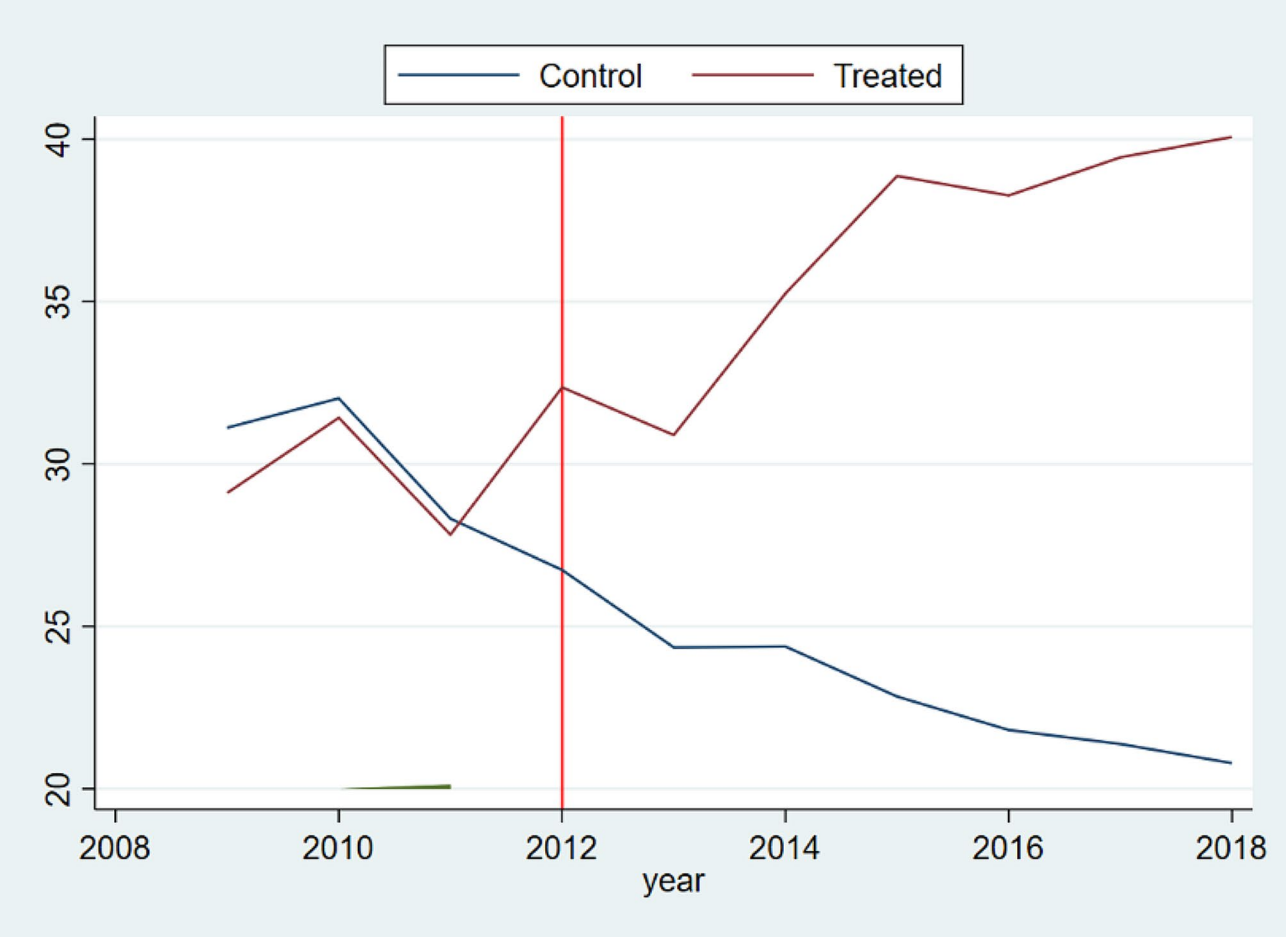
Trend in PHC Outpatient Market Share (2009–2018). Notes: This graph shows the trend in PHC Outpatient Market Share from 2009 to 2018, with clear labels on the vertical axis for the percentage of outpatient visits at PHC facilities and on the horizontal axis for the years. The vertical red line indicates the timing of the financing reform in 2012

Between 2009 and 2018, the percentage of outpatients accessing initial care in rural community health centres or township health centres in Pengshui County rose from 29% (522,700 out of 1,817,600) in 2009 to 40% (849,900 out of 2,147,800) in 2018, compared to a national average of 23% in 2018 [23]. Figure 3 depicts the trend in the proportion of outpatient visits at rural PHC facilities in Pengshui County and its synthetic control. In the pre-reform period, the outcomes were nearly identical, furnishing a plausible counterfactual for Pengshui without the integrated financing model. After 2012, the proportion declined steadily for the synthetic control but rose for Pengshui, indicating the reform improved PHC’s care-seeking preference. The gap widened through 2018.

The SDID model estimates that the reform increased the percentage of outpatient visits at PHC facilities in Pengshui by 14.92%(95% confidence interval, CI: 6.59–23.24)compared to the synthetic control, indicating that PHC centres are slowly regaining their appeal. The effect is significant at the 1% level (Table 4). Thus, Hypothesis 1 is substantially supported by the evidence.

**Table 4:** SDID estimates of the average treatment effects

	Percentage of outpatient visits at PHC facilities	Per capita total PHC expenditure
ATT	14.92^***^	87.30^**^
	(4.24)	(42.64)

Notes: ATT = Average Treatment Effects on Treated; PHC = Primary Health Care. Results indicate the reform substantially increased the percentage of outpatient visits at PHC facilities by 14.92% while raising per capita PHC expenditure by 87.30 CNY. Standard errors (in parentheses) are estimated using the placebo method. Significance levels: ***1%; **5%; *10%

#### Trends in PHC expenditure market share

**Fig. 4:**
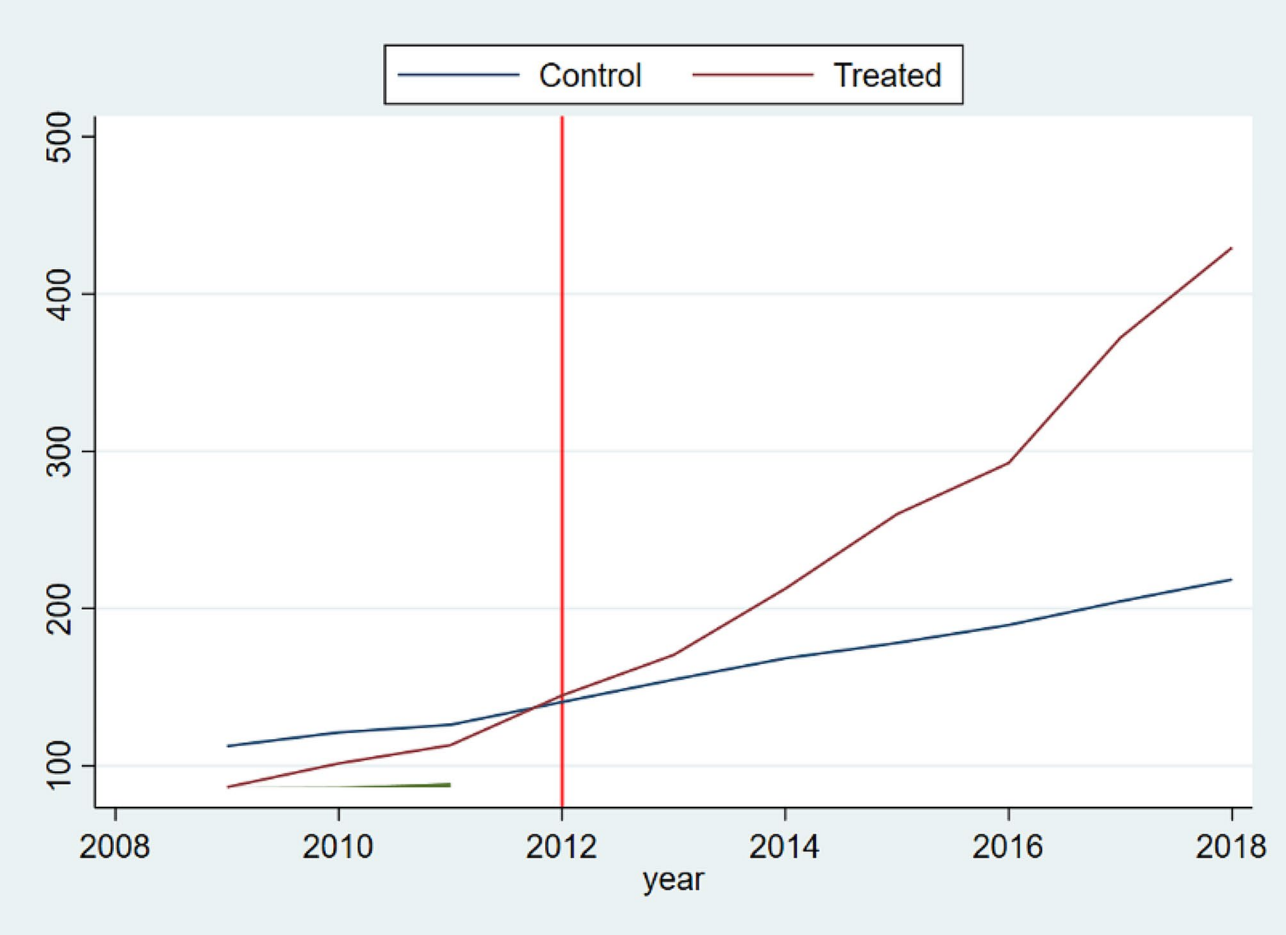
Trend in per capita total PHC expenditure (2009–2018). Notes: This graph illustrates the trend in Per Capita total PHC Expenditure from 2009 to 2018, with labels on the vertical axis for per capita expenditure on PHC (in CNY) and on the horizontal axis for the years. The vertical red line indicates the timing of the financing reform in 2012

Between 2009 and 2018, annual per capita total PHC expenditure increased from 86.41 CNY to 429.44 CNY in Pengshui County [22]. Figure 4 shows trends in per capita health spending flowing to PHC facilities. In the pre-reform period, the series were similar between Pengshui and its synthetic control. After 2012, Pengshui’s PHC spending rose while the control group remained relatively flat. The gap widened through 2018.

The average treatment effect represents the reform increased annual per capita PHC spending by 87.30 CNY (95% CI:3.71-170.88). The estimate is significant at the 5% level (Table 4). This provides evidence that the pooling arrangement channelled more health resources toward township and community health service centres. Accordingly, Hypothesis 2 receives substantial support.

While our study demonstrates an increase in PHC utilization following the implementation of the Pengshui model, we hypothesize that enhancing PHC and boosting its utilization could potentially mitigate unnecessary higher-level hospitalizations and enhance the overall efficiency of healthcare resource utilization. However, although the number of inpatients in primary medical institutions tripled from 2009 to 2018, the proportion of inpatients in PHC decreased slightly from 67% (30,528 out of 45,450) to 60% (93,273 out of 156,166) [23]. This outcome indicates that a comprehensive shift away from hospital-centric to PHC-based healthcare necessitates sustained efforts to bolster the capacity of primary care, particularly in inpatient and specialist services.

### Dynamic effects of the reform

To further investigate the temporal impact of the financing reform, we estimated the average treatment effects on the treated (ATT) for each year following the 2012 reform, from 2013 to 2018. The results, as depicted in Table 5, reveal a gradual and sustained increase in both the percentage of outpatient visits at PHC facilities and per capita total PHC health expenditures over the years. The reform’s impact on the percentage of outpatient visits at PHC facilities shows a significant and increasing trend over time, reaching 14.92% (*p* < 0.01) by 2018. Additionally, the ATT for per capita total PHC expenditure also exhibits a year-on-year increase, with a significant rise of 87.30 CNY (*p* < 0.05) by 2018.

**Table 5:** Dynamic ATT of the financing reform on PHC utilization and expenditures, 2013–2018

	Percentage of outpatient visits at PHC facilities	Per capita total PHC expenditure
2013	6.70	16.42
	(4.14)	(26.53)
2014	8.54^*^	26.80
	(4.24)	(33.03)
2015	10.62^**^	40.22
	(4.24)	(35.95)
2016	12.04^***^	51.11
	(4.38)	(40.17)
2017	13.12^***^	67.63
	(4.42)	(41.22)
2018	14.92^***^	87.30^**^
	(4.24)	(42.64)

Notes: ATT = Average Treatment Effects on Treated; PHC = Primary Health Care. Standard errors (in parentheses) are estimated using the placebo method. Significance levels: ***1%; **5%; *10%

These findings suggest that the financing reform had a progressive and long-lasting impact on PHC utilization and resource allocation, with the effects becoming more pronounced and statistically significant over time. This outcome aligns with our expectations that the benefits of the reform would become more evident as the initiatives are fully implemented and absorbed by the local health system. The dynamic analysis highlights the importance of considering the temporal dimension when evaluating the impact of health financing interventions, as the full effects may take several years to materialize. These findings underscore the importance of sustaining policy commitment and financing reforms over an extended time horizon to realize the transformative potential of integrated PHC models. By maintaining a long-term perspective and providing consistent support, policymakers can create an enabling environment for PHC financing reforms to succeed and generate sustainable improvements in population health.

### Placebo and robustness tests

In our study, we implemented a placebo test in space by alternately designating each of the 37 counties in the region as the treated county instead of Pengshui County. This approach aimed to reassess the model to determine varying treatment effects. Our findings indicated that most counties did not exhibit significant effects at critical levels of 5% and 1%, except for two urban areas—Jiulongpo and Nanan districts. For the variable concerning the proportion of outpatient visits, both Jiulongpo and Nanan Districts exhibited significance at the 10% level. However, regarding primary healthcare funding, Jiulongpo District did not show significance, while Nanan District demonstrated significant effects at the 5% level. This nuanced outcome underscores the distinctiveness of Pengshui’s case, where the magnitude of its estimates surpassed those of most other counties and districts (see Supplementary Table 1).

To further ensure the robustness of our results, we employed several methodologies. One method involved using an alternative synthetic control, yielding consistent outcomes with our initial findings (see Supplementary Fig. 2). Another approach restricted the control group to only 5 neighbouring counties of Pengshui County, based on the rationale that neighbouring regions likely share similar economic, social, and demographic characteristics. This modification still resulted in consistent treatment effects. Furthermore, we assessed the impact of exclusively including rural counties in the synthetic control group, excluding 9 urban areas, due to their socio-economic similarities with Pengshui County. This assessment also supported the initial results, thereby reinforcing the validity and robustness of our conclusions. The detailed data pertaining to these tests and methodologies are presented in Supplementary Table 2. These tables provide a comprehensive view of the SDID estimates of the average treatment effects for various control groups, along with the standard errors and significance levels for each estimate.

### Mechanism analysis

The reform’s impact on outpatient visits and per capita PHC expenditure was further investigated to understand the underlying mechanisms. We identified two key indicators of PHC capacity: the number of inpatient beds per 1,000 population and the number of health technicians per 1,000 population in PHC facilities. These indicators were selected based on their relevance to the accessibility and quality of PHC services, which are critical factors influencing patients’ care-seeking behavior and trust in primary care providers [12, 15]. The number of inpatient beds per 1,000 population reflects the availability of infrastructure and resources to meet the population’s healthcare needs, while the number of health technicians per 1,000 population indicates the workforce capacity to deliver quality PHC services [28]. The SDID estimates revealed a significant increase in both indicators post-reform, with 0.49 additional inpatient beds per 1,000 population (*p* < 0.10) and 0.33 additional health technicians per 1,000 population (*p* < 0.05) in Pengshui County compared to the synthetic control(Table 6). These findings indicate that the financial reform contributed to the enhancement of PHC infrastructure and workforce capacity.

**Table 6:** ATT of the financing reform on PHC capacity indicators

	Number of inpatient beds per 1,000 population	Number of health technicians per 1,000 population
ATT	0.49^*^	00.33^**^
	(0.25)	(0.13)

Notes: ATT = Average Treatment Effects on Treated. Standard errors (in parentheses) are estimated using the placebo method. Significance levels: ***1%; **5%; *10%

## Discussion

### Implications of mechanisms analysis

The integration of financial resources and the strategic allocation of funds, as evidenced by the increased number of inpatient beds and health technicians, underscore the reform’s effectiveness in addressing the accessibility and quality of PHC services. These findings suggest that the reform’s success in increasing PHC utilization and expenditures can be partly attributed to the expansion of PHC infrastructure and workforce. Specifically, government investments and the establishment of an integrated fund addressed issues of fragmented resources and inadequate funding, enabling PHC institutions to provide higher-quality services. The additional resources channelled to PHC facilities through the integrated financing mechanism allowed for investments in inpatient beds and the recruitment of more health technicians, thereby improving access to primary care services. Moreover, the reform improved the recruitment and retention of medical personnel by increasing their income and providing more training opportunities. This not only enhanced the quality of medical services but also increased residents’ trust in PHC institutions, thereby promoting greater utilization of PHC services. Furthermore, horizontal integration and centralized procurement mechanisms increased the efficiency of drug and equipment procurement, reducing operational costs. This allowed PHC institutions to better manage their limited resources and allocate more funds to critical service areas. Thus, the integrated financing mechanism, performance-based incentives, and centralized management likely worked together to channel resources towards PHC infrastructure and workforce improvements, ultimately making PHC more accessible, affordable, and attractive to patients.

### Interpretations

The innovative reform in Pengshui provides an informative case study of how horizontal financial integration can strengthen PHC systems and improve access in underserved rural areas. Drawing on Ostrom’s design principles for sustainable management of common pool resources [32], several key features of Pengshui’s approach are instructive for most resource-constrained settings confronting fragmented funding and delivery systems.

First, the implementation of dedicated fund pooling addressed longstanding underinvestment by transforming the revenues of PHC providers into a common property resource system with well-defined boundaries, rights, and governance protocols [33]. Unlike certain low- and middle-income nations dependent on external financial assistance [34, 35], this pooling mechanism emphasizes internal cross-subsidization within the provider. In Pengshui County, the innovative capital pool substantially offsets financial investment deficits, notwithstanding the economic development constraints of the region. This novel approach proved critical in overcoming challenges related to sustainable investment, culminating in significant improvements in the infrastructure and facilities of primary medical institutions. Moreover, it facilitated the development and recruitment of medical professionals, thereby playing a crucial role in enhancing the service capabilities and quality of primary healthcare institutions. This strategic financial innovation not only mitigated the investment constraints but also laid a foundation for a more resilient and efficient primary healthcare system in the low-resource region.

Second, the horizontal pooling arrangement played a crucial role in bolstering equity within primary care services. This system effectively mitigated tendencies for free-riding and opportunistic behaviour, typical pitfalls in voluntary contribution schemes. Acknowledging global fiscal constraints, it was imperative to manage pooled public funds with utmost efficiency and equity [36]. Mandatory participation, alongside revenue-based contributions, ensured that wealthier facilities contributed more, subsidizing counterparts in less affluent or remote regions. The cross-subsidization mechanism not only equalized resource distribution but also conformed to the principle of needs-based allocation, directing resources to areas of greatest need. Consequently, the rule of proportional contributions was instrumental in fostering fairness and a cooperative spirit among various facilities towards constructing a unified and equitable PHC system. This approach enabled Pengshui to establish a framework wherein facilities, irrespective of financial status, contributed to and benefited from a more equitable and accessible primary care system.

Third, this innovative funding model exemplifies the principles of investing more and investing better in PHC [4]. The pooling model facilitated a more equitable distribution of resources, prioritizing health needs over mere local revenue generation. The inclusion of a carryover provision in this model promoted long-term investment and strategic planning. Pooling resources from various organizations enabled a fairer fund distribution, placing greater emphasis on health requirements rather than the revenue-generating capacities of local areas. This method effectively countered resource fragmentation, directing scarce funds towards major hubs in areas with high population density and significant health demands. The enhanced purchasing power of the collective pool facilitated strategic purchasing, aligning resource distribution with system-wide health priorities rather than the isolated needs of individual providers [10]. This strategic and comprehensive investment in PHC not only improved care quality but also guaranteed a more sustainable and effective response to primary healthcare challenges.

Fourth, the horizontally structured pooling model markedly improved the efficiency of governmental administration in primary healthcare. This model acted as a pivotal element in fortifying the synergy among diverse primary medical institutions within the PHCIG, enhancing group unity. Centralized procurement of pharmaceuticals and medical consumables via the capital pool escalated purchase volumes, thereby enhancing negotiating leverage. Furthermore, the introduction of oversight and audit systems for the capital pool augmented transparency in financial management. This approach not only guaranteed the secure and efficient utilization of pooled funds but also bolstered governance through systematic monitoring and graduated sanctions to ensure compliance. Collectively, these initiatives contributed to a more streamlined, transparent, and efficient resource management within primary healthcare. Simultaneously, with the support of PHC funds, telemedicine and two-way referrals have been facilitated through the upgrading of the medical information-sharing system. This has strengthened vertical collaboration between PHC institutions and higher-level hospitals. As a result, vertical collaboration is reinforced, collectively enhancing the overall efficiency of the county medical service system.

### Policy implications

As countries rebuild from COVID-19, integrated financing policies take on heightened urgency to reinforce PHC resilience against future shocks [4, 37]. The Pengshui model demonstrates an effective health financing approach to extend access to underserved populations and withstand crises. Our findings suggest several implications:

The increase in PHC outpatient utilization implies that financing integration and governance changes redirected patient care-seeking preferences towards primary facilities. This indicates that the model improved perceptions of PHC quality and trust. Policymakers should consider reforms to unify governance and integrate financing channels as a means of rebuilding foundational primary care capacities. The rise in per capita total PHC expenditures shows that pooling and centralized purchasing enabled a more needs-based allocation of scarce resources. Policymakers can apply this approach to improve equity and strategic investment tailored to local priorities. Legal frameworks may be needed to mandate contributions and oversight.

The reform in Pengshui County provides an informative case study for other resource-constrained settings on utilizing supply-side financing integration to strengthen PHC. However, implementing integrated pooling faces challenges around institutional constraints, resistance from better-resourced facilities fearing cross-subsidization, inadequate purchasing capabilities, and underdeveloped legal frameworks [10]. Our findings suggest that even incremental integration steps can generate efficiency and potentially contribute to improved equity in access to PHC services. Policymakers should actively evaluate reforms even if conducted sequentially or on a limited scale. The Pengshui model demonstrates an effective health financing approach to progressively realize universal coverage given fiscal constraints.

This study has several limitations. First, only one treated county was analyzed as the reform has not been widely replicated. Future research could involve larger groups of counties using comparative case studies. Secondly, estimated financial impacts may be conservative, as increased outpatient utilization could raise subsequent inpatient and drug expenditures. Further investigation into these dynamic effects is warranted. Thirdly, utilization alone does not guarantee improved care, thus quality impacts require investigation. Additionally, changes in out-of-pocket spending may reflect shifts in patient behavior, warranting disaggregation of reform effects on government budgets and patient costs. Lastly, future research should prioritize the collection and analysis of detailed data on PHC fund allocation and usage. This should encompass key service delivery indicators such as drug prices across different procurement models, workforce competencies, equipment availability, patient care-seeking preferences and satisfaction. However, we acknowledge that the scope of our study was limited to examining the direct impact of horizontal integration on PHC utilization and expenditure. Future research should prioritize a more comprehensive investigation of how the Pengshui model affects the entire rural healthcare system, including its influence on referral patterns, care coordination, and health outcomes across different levels of care.

## Conclusions

This study provides robust evidence that horizontal integration and resource-pooling in PHC financing improved service delivery and utilization in rural China. The model’s emphasis on compulsory contributions, strategic purchasing, and performance incentives demonstrates an effective health financing strategy to promote access and equity in rural China, with potential lessons for most resource-constrained settings. By bridging evidence gaps constraining PHC financing policy, integrated financing offers a pathway to reinforce primary care capacities and progress towards UHC.

### Electronic supplementary material

Below is the link to the electronic supplementary material.

Supplementary Material 1

Supplementary Material 2

## Data Availability

The datasets used and/or analysed during the current study are available from the corresponding author on reasonable request.

## Abbreviations

ATT: Average Treatment Effects on Treated
CI: Confidence Interval
CPHC: Commission of Primary Health Care
DID: Difference-in-Differences
GDP: Gross Domestic Product
LMICs: Low- and Middle-Income Countries
PHC: Primary Health Care
PHCIG: Primary Health Care Institution Group
RHMC: Rural Health Management Centre
SCM: Synthetic Control Method
SDID: Synthetic Difference-in-Differences
UHC: Universal Health Coverage
WHO: World Health Organization

## References

[1] Universal health coverage (UHC). [Internet]. [cited 2024 Jan 18]. https://www.who.int/news-room/fact-sheets/detail/universal-health-coverage-(uhc).

[2] Kruk ME, Gage AD, Arsenault C, Jordan K, Leslie HH, Roder-DeWan S (2018). High-quality health systems in the Sustainable Development goals era: time for a revolution. Lancet Glob Health.

[3] Organization WH, Kutzin J, Witter S, Jowett M, Bayarsaikhan D. Developing a national health financing strategy: a reference guide [Internet]. World Health Organization; 2017 [cited 2023 Oct 7]. https://iris.who.int/handle/10665/254757.

[4] Hanson K, Brikci N, Erlangga D, Alebachew A, De Allegri M, Balabanova D (2022). The Lancet Global Health Commission on financing primary health care: putting people at the centre. Lancet Global Health.

[5] World Health Organization, McIntyre D, Kutzin J. Health financing country diagnostic: a foundation for national strategy development [Internet]. Geneva: World Health Organization; 2016 [cited 2023 Oct 11]. 50 p. (Health Financing Diagnostics;1). https://iris.who.int/handle/10665/204283.

[6] Chu A, Kwon S, Cowley P (2019). Health Financing reforms for moving towards Universal Health Coverage in the Western Pacific Region. Health Syst Reform.

[7] D’Aquino L, Pyone T, Nigussie A, Salama P, Gwinji G, Van Den Broek N (2019). Introducing a sector-wide pooled fund in a fragile context: mixed-methods evaluation of the health transition fund in Zimbabwe. BMJ Open.

[8] Hoa NT, Derese A, Peersman W, Markuns JF, Willems S, Tam NM. Primary care quality in Vietnam: Perceptions and opinions of primary care physicians in commune health centers – a mixed-methods study. Vaingankar JA, editor. PLoS ONE. 2020;15(10):e0241311. 10.1371/journal.pone.0241311 PMC7595414 33119666

[9] Lim MY, Kamaruzaman HF, Wu O, Geue C (2023). Health financing challenges in Southeast Asian countries for universal health coverage: a systematic review. Arch Public Health.

[10] Mathauer I, Vinyals Torres L, Kutzin J, Jakab M, Hanson K (2020). Pooling financial resources for universal health coverage: options for reform. Bull World Health Organ.

[11] Mathauer I, Saksena P, Kutzin J (2019). Pooling arrangements in health financing systems: a proposed classification. Int J Equity Health.

[12] Li X, Lu J, Hu S, Cheng K, De Maeseneer J, Meng Q (2017). The primary health-care system in China. Lancet.

[13] Babiarz KS, Miller G, Yi H, Zhang L, Rozelle S (2010). New evidence on the impact of China’s New Rural Cooperative Medical Scheme and its implications for rural primary healthcare: multivariate difference-in-difference analysis. BMJ.

[14] Yip WCM, Hsiao WC, Chen W, Hu S, Ma J, Maynard A (2012). Early appraisal of China’s huge and complex health-care reforms. Lancet.

[15] Yip W, Fu H, Chen AT, Zhai T, Jian W, Xu R (2019). 10 years of health-care reform in China: progress and gaps in Universal Health Coverage. Lancet.

[16] Tao W, Zeng Z, Dang H, Li P, Chuong L, Yue D (2020). Towards universal health coverage: achievements and challenges of 10 years of healthcare reform in China. BMJ Glob Health.

[17] Li C, Chen Z, Khan MM (2021). Bypassing primary care facilities: health-seeking behavior of middle age and older adults in China. BMC Health Serv Res.

[18] Zhou Z, Zhao Y, Shen C, Lai S, Nawaz R, Gao J (2021). Evaluating the effect of hierarchical medical system on health seeking behavior: a difference-in-differences analysis in China. Soc Sci Med.

[19] Yu H (2015). Universal health insurance coverage for 1.3 billion people: what accounts for China’s success?. Health Policy.

[20] Wang H, Zhang L, Yip W, Hsiao W (2011). An experiment in payment reform for doctors in rural China reduced some unnecessary Care but did not lower total costs. Health Aff.

[21] Yip W, Fu H, Jian W, Liu J, Pan J, Xu D (2023). Universal health coverage in China part 2: addressing challenges and recommendations. Lancet Public Health.

[22] Zeng Z, Tao W, Ding S, Fang J, Wen J, Yao J (2022). Horizontal integration and financing reform of rural primary care in China: a model for low-resource and remote settings. IJERPH.

[23] Zeng Z, Tao W, Liu C, Zhou L, Pan J, Zhang W (2019). Horizontal integration and financial reform of a primary care delivery system in Pengshui County, China: a case study. Lancet.

[24] Abadie A, Diamond A, Hainmueller J (2010). Synthetic control methods for comparative Case studies: estimating the Effect of California’s Tobacco Control Program. J Am Stat Assoc.

[25] Arkhangelsky D, Athey S, Hirshberg DA, Imbens GW, Wager S (2021). Synthetic difference-in-differences. Am Econ Rev.

[26] Pengshui Miao and Tujia Autonomous County. In: Wikipedia [Internet]. 2021 [cited 2022 May 5]. https://en.wikipedia.org/w/index.php?title=Pengshui_Miao_and_Tujia_Autonomous_County&oldid=1055532271

[27] Jamison DT, Summers LH, Alleyne G, Arrow KJ, Berkley S, Binagwaho A (2013). Global health 2035: a world converging within a generation. Lancet.

[28] Primary health care measurement framework. and indicators: monitoring health systems through a primary health care lens [Internet]. [cited 2022 Jun 12]. https://www.who.int/publications-detail-redirect/9789240044210.

[29] Dovern J, Frank J, Glas A, Müller LS, Perico Ortiz D (2023). Estimating pass-through rates for the 2022 tax reduction on fuel prices in Germany. Energy Econ.

[30] Abman R, Longbrake G (2023). Resource development and governance declines: the case of the Chad–Cameroon petroleum pipeline. Energy Econ.

[31] Bertrand M, Duflo E, Mullainathan S (2004). How much should we trust Differences-In-Differences estimates?. Q J Econ.

[32] Ostrom E. Governing the commons. Cambridge University Press; 2015. p. 297.

[33] Ostrom E (2000). Collective action and the evolution of social norms. J Economic Perspect.

[34] Ma-Nitu SM (2022). Financing primary health care: seeing the bigger picture. Lancet Global Health.

[35] Moosa S (2022). Provider perspectives on financing primary health care for universal health coverage. Lancet Global Health.

[36] Brundtland GH (2022). Public financing for primary health care is the key to universal health coverage and strengthening health security. Lancet Global Health.

[37] Kurowski C, Evans DB, Tandon A, Eozenou PHV, Schmidt M, Irwin A et al. From Double Shock to Double Recovery: Implications and Options for Health Financing in the Time of COVID-19. 2021 Mar [cited 2023 Oct 6]; http://hdl.handle.net/10986/35298.

