# Supplementary material for: Improving access to primary health care through financial innovation in rural China: a quasi-experimental synthetic difference-in-differences approach
Source: BMC Prim Care. 2024 Jun 1;25:195. doi: 10.1186/s12875-024-02450-0 (PMC11143622; doi:10.1186/s12875-024-02450-0)
Supplement: Supplementary file 1 — Supplementary Material 1 [file 12875_2024_2450_MOESM1_ESM.docx]

**
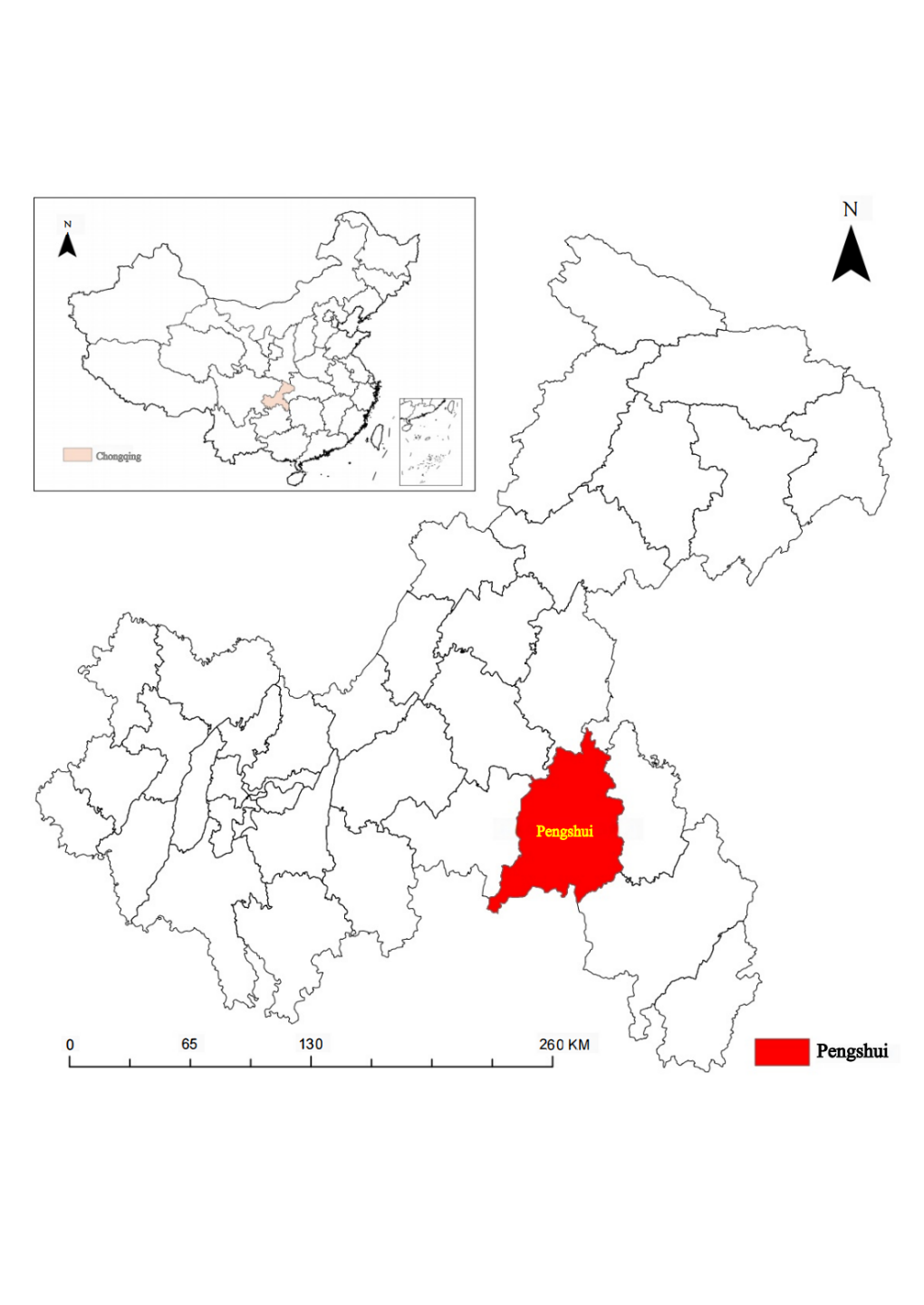
**

**Supplementary Figure 1.** The location of Pengshui County, Chongqing, China.


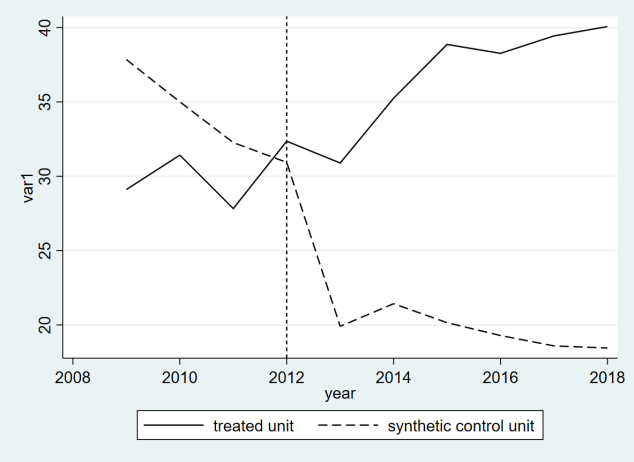

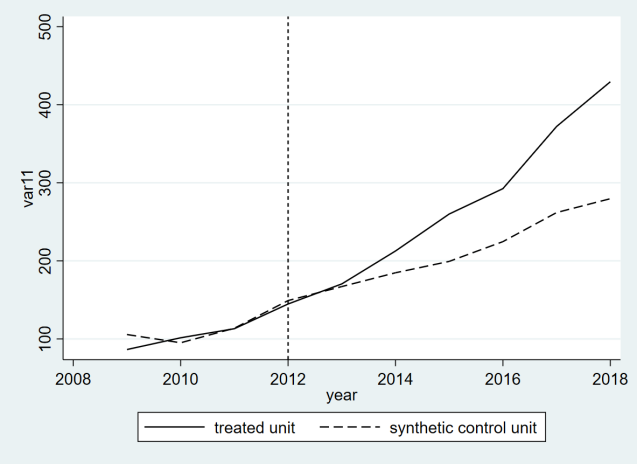


(a) (b)

**Supplementary Figure 2.** Alternative synthetic control estimates (SCM).

Notes: The solid line(a) shows outpatient market share in PHC trend of Pengshui from 2009 to 2018. By comparison, the dashed line shows the synthetic control group based on the SCM. The solid line(b) shows per capita PHC spending trend of Pengshui from 2009 to 2018, while the dashed line shows the synthetic control group based on the SCM. The vertical dotted line indicates the timing of the financing reform in 2012.
