# Supplementary material for: Improving access to primary health care through financial innovation in rural China: a quasi-experimental synthetic difference-in-differences approach
Source: BMC Prim Care. 2024 Jun 1;25:195. doi: 10.1186/s12875-024-02450-0 (PMC11143622; doi:10.1186/s12875-024-02450-0)
Supplement: Supplementary file 2 — Supplementary Material 2 [file 12875_2024_2450_MOESM2_ESM.docx]

**Supplementary Table 1.** SDID estimates of the average treatment effects (Placebo test)

| County/District | 1 | 2 | 3 | 4 | 5 | 6 | 7 | 8 | 9 | 10 |
| --- | --- | --- | --- | --- | --- | --- | --- | --- | --- | --- |
| Percentage of outpatient visits at PHC facilities | 2.75  （4.41） | -5.98  （4.35） | 1.35  （4.67） | 2.92  （4.51） | -2.01  （4.46） | 7.17 （4.39） | **7.78*  （4.19）** | **5.21*  （4.42）** | 5.21  （4.42） | 1.58  （4.45） |
| Per capita total PHC expenditure | 17.36  （42.35） | -60.30 （41.62） | 47.01  （43.99） | -24.75  （41.67） | -8.80  （42.06） | 58.56  （43.09） | -37.04 （40.77） | **90.13**  （36.63）** | 22.16 （42.22） | 6.42 （41.62） |
| County/District | 11 | 12 | 13 | 14 | 15 | 16 | 17 | 18 | 19 | 20 |
| Percentage of outpatient visits at PHC facilities | 0.62  （4.50） | -9.10 （4.10) | 3.42  (4.41) | -4.74  (4.29) | 2.65  (4.52) | -4.58  (4.26) | -0.53 (4.52) | -6.66  (4.39) | -4.37  (4.35) | -3.56  (4.48) |
| Per capita total PHC expenditure | 65.19 （42.02） | -74.55   (39.22) | 48.34  (41.43) | -3.09   (42.13) | -42.74  (41.73) | 25.50 (42.42) | 19.29 (42.52) | 8.73 (42.02) | 12.95  (42.20) | -38.40  (42.99) |
| County/District | 21 | 22 | 23 | 24 | 25 | 26 | 27 | 28 | 29 | 30 |
| Percentage of outpatient visits at PHC facilities | 1.26 (4.41) | 8.11 (4.28) | -1.18  (4.54) | -2.09 (42.90) | -3.58 (4.35) | 1.70 (4.49) | 3.3 (4.71) | -0.29 (4.38) | -1.98 (4.48) | 7.07  (4.35) |
| Per capita total PHC expenditure | -51.06 (40.86) | 11.36 (42.03) | -2.09 (42.90) | -3.38 (42.61) | 2.75 (42.66) | -37.25 (41.45) | 8.65 (42.93) | -57.41 (40.72) | 21.53  (42.47) | 11.75  (42.48) |
| County/District | 31 | 32 | 33 | 34 | 35 | 36 | 37 | 38 |  |  |
| Percentage of outpatient visits at PHC facilities | 2.80 (4.40) | -6.76  (4.30) | 6.06 (4.35) | -9.05 (4.21) | 0.59 (4.60) | -9.83 (4.48) | 2.62 (4.38) | **14.92*** (4.24)** |  |  |
| Per capita total PHC expenditure | 42.36 (41.45) | -4.60  (41.90) | -9.87 (42.33) | -56.93 (42.80) | -16.30 (43.00) | -9.95 (42.05) | 37.30 (41.87) | **87.3** (42.64)** |  |  |

Notes: PHC=Primary Health Care. Standard errors in parentheses. Jiulongpo District (Area 7), Nanan District (Area 8), and Pengshui County (Area 38). Significance levels: ***1%; **5%; *10%.

**Supplementary Table 2.** SDID estimates of the average treatment effects of Pengshui County (Robust test)

|  | Percentage of outpatient visits  at PHC facilities | Per capita total PHC expenditure |
| --- | --- | --- |
| The control group consisted only of Pengshui County’s 5 neighbouring counties | 18.21^**^ | 84.13^*^ |
|  | (7.89) | (51.19) |
| The control group comprised 28 rural counties, excluding 9 urban areas. | 15.26^***^ | 81.95^**^ |
|  | (4.75) | (34.98) |

Notes: ATT=Average Treatment Effects on Treated; PHC=Primary Health Care. Standard errors in parentheses. Significance levels: ***1%; **5%; *10%
